# Supplementary material for: Changes in countermovement jump force-time characteristic in elite male basketball players: A season-long analyses
Source: PLoS One. 2023 Sep 27;18(9):e0286581. doi: 10.1371/journal.pone.0286581 (PMC10529540; doi:10.1371/journal.pone.0286581)
Supplement: Attachment — Submitted filename: PlosOne Response to Reviewers.docx [file pone.0286581.s001.docx]

December 30^th^, 2022

Editors and Reviewers, PlosOne Journal

RE: PONE-D-22-31306

Dear Editors and Reviewers,

Thank you for critically reviewing our article, “Changes in Countermovement Jump Force-Time Characteristic in Elite Male Basketball Players: A Season-Long Analyses”.

.

Reviewers have given authors important considerations resulting in changes and the manuscript is better based on the changes that have come as a result.

For simplicity, we have attached an updated manuscript with all changes highlighted in blue. You will find that we have reworked several paragraphs directly considering constructive comments by reviewer #2. If more changes are needed, please let us know.

Thank you for your consideration of our manuscript. Authors look forward to future correspondence regarding acceptability for publication.

Sincerely,

Research Team

**Specific Comments and Author Response for Reviewer #1:**

***Line 46-47***

“Need a reference”.

*Authors have added a reference to the respective location*. *Thank you for your constructive feedback.*

***Line 71-73***

“I suggest updating the definition.”

*Dear reviewer, thank you for your comment. We have eliminated our definition and have cited a definition from the article proposed by yourself.*

***Table 1***

“The table is not clear, the 57 evaluations correspond to the evaluations of the 12 athletes participating in the study during 7 days of testing? if so, shouldn't it be 84?
and likewise for the other periods. I suggest an explanatory or explanatory note at the bottom of the table”

*We have added a sentence in the paragraph above to clarify what is meant by test days and athlete screenings to avoid any potential confusion. We hope this clarifies things. Given the real-world nature of our data, not all 12 athletes performed the jumps on every single test day due to various reasons (e.g., sickness, class conflicts etc.). We account for these “holes” in the data by using linear mixed effect models as our statistical approach.*

***Line 122***

“Mention the code.”

Thank you, we have added the respective IRB approval number.

***Line 142***

***“***This is repeating what is already in table 1”.

*Dear reviewer, thank you for your constructive feedback. We have eliminated the respective sentence since the information is already provided in table 1.*

**Line 156**

**“**I suggest a quote that supports this decision”.

*Dear reviewer, thank you for your comment, we have added an addition to the sentence, as well as the citation.*

**Figures**

the quality of the images is not the best, try to improve the resolution

*Dear reviewer, we believe the quality of the images (JPEG) that were initially submitted are of good quality, however the quality might have been influence in the conversion to a PDF. In this case we would like to refer to the editor for a final decision. If indeed the quality of our images needs improvement, we’d be happy to look into this and make respective changes.*

Thank you, Reviewer #1, for your comments and suggestions. By addressing your prompts, the manuscript should better appeal to readership.

**Specific Comments and Author Response for Reviewer #2:**

**LN 54-66**

“Re-organize in a way that it highlights the changes in jump mechanics across training phases. Use the lack of studies in real-world settings in the last part to justify the need for this kind of study”

*Dear reviewer, thank you for your constructive feedback. We have reordered the paragraph in a way where now, the respective studies are mentioned in the beginning of the paragraph, and the rationale behind why studies in real-world settings are needed, towards the end of the paragraph. We hope this paragraph now reads clearer.*

**LN 67-69**

“Paraphrase into more scientific stream”.

*Highlighted in blue, we have made respective changes to the proposed location.*

**LN 70**

“Change hopping to jumping”

*Thank you, we have made the proposed change.*

**LN 78-80**

“Just use force platform from this point onwards. Integrate LN 80-81.”

*Dear reviewer, thank you for this comment, we have made the proposed change in our manuscript.*

**LN 125**

“Is the dynamic warm-up consistent across training phases?

*Dear reviewer, thank you for your constructive feedback. We have added a note clarifying that the warmup procedures stayed consistent over the duration of the study.*

**LN 208-260**

“Too much information presented here. Breakdown into two paragraphs (1: Key findings; 2: Rationale for results).”

*Dear reviewer, thank you for your comment. We have reduced our discussion, to make it more concise. However, in line with PlosOne’s guidelines, especially in regard to interpreting the results, and how they relate to the hypothesis, and previously conducted research, we fear that by eliminating further sections from the discussion, our manuscript loses some strength. If in its current standing, the discussion section still does not fulfill what is expected for publication, we kindly ask you, as well as the editorial team to make suggestions slightly more detailed, in order for us authors to edit specific parts of the discussion. We’d be happy to include further changes if needed, and greatly appreciate your feedback and believe our manuscript will be improved based on your comments.*

**REFERENCES**

*Thank you, we have made suggested changes to our references, and highlighted these in blue.*

Thank you, Reviewer #2, for your comments and suggestions. By addressing your prompts, the manuscript should better appeal to readership.
